# *Treponema pallidum* Infection in the Wild Baboons of East Africa: Distribution and Genetic Characterization of the Strains Responsible

**DOI:** 10.1371/journal.pone.0050882

**Published:** 2012-12-20

**Authors:** Kristin N. Harper, Robert D. Fyumagwa, Richard Hoare, Philemon N. Wambura, Dorian H. Coppenhaver, Robert M. Sapolsky, Susan C. Alberts, Jenny Tung, Jeffrey Rogers, Morris Kilewo, Emmanuel K. Batamuzi, Fabian H. Leendertz, George J. Armelagos, Sascha Knauf

**Affiliations:** 1 Department of Environmental Health Sciences, Columbia University Medical Center, New York, New York, United States of America; 2 Wildlife Veterinary Program, Tanzania Wildlife Research Institute, Arusha, Tanzania; 3 Department of Veterinary Microbiology and Parasitology, Faculty of Veterinary Medicine, Sokoine University of Agriculture, Morogoro, Tanzania; 4 Department of Microbiology and Immunology, University of Texas Medical Branch, Galveston, Texas, United States of America; 5 Biological Sciences, Stanford University, Stanford, California, United States of America; 6 Institute of Primate Research, National Museums of Kenya, Nairobi, Kenya; 7 Department of Biology, Duke University, Durham, North Carolina, United States of America; 8 Department of Evolutionary Anthropology, Duke University, Durham, North Carolina, United States of America; 9 Population Research Institute, Duke University, Durham, North Carolina, United States of America; 10 Human Genome Sequencing Center, Baylor College of Medicine, Houston, Texas, United States of America; 11 Department of Ecology, Tanzania National Parks, Arusha, Tanzania; 12 Department of Veterinary Surgery and Theriogenology, Sokoine University of Agriculture, Morogoro, Tanzania; 13 Research Group Emerging Zoonoses, Robert Koch-Institute, Berlin, Germany; 14 Department of Anthropology, Emory University, Atlanta, Georgia, United States of America; 15 Pathology Unit, German Primate Centre, Leibniz Institute for Primate Research, Göttingen, Germany; Columbia University, United States of America

## Abstract

It has been known for decades that wild baboons are naturally infected with *Treponema pallidum*, the bacterium that causes the diseases syphilis (subsp. *pallidum*), yaws (subsp. *pertenue*), and bejel (subsp. *endemicum*) in humans. Recently, a form of *T. pallidum* infection associated with severe genital lesions has been described in wild baboons at Lake Manyara National Park in Tanzania. In this study, we investigated ten additional sites in Tanzania and Kenya using a combination of macroscopic observation and serology, in order to determine whether the infection was present in each area. In addition, we obtained genetic sequence data from six polymorphic regions using *T. pallidum* strains collected from baboons at two different Tanzanian sites. We report that lesions consistent with *T. pallidum* infection were present at four of the five Tanzanian sites examined, and serology was used to confirm treponemal infection at three of these. By contrast, no signs of treponemal infection were observed at the six Kenyan sites, and serology indicated *T. pallidum* was present at only one of them. A survey of sexually mature baboons at Lake Manyara National Park in 2006 carried out as part of this study indicated that roughly ten percent displayed *T. pallidum*-associated lesions severe enough to cause major structural damage to the genitalia. Finally, we found that *T. pallidum* strains from Lake Manyara National Park and Serengeti National Park were genetically distinct, and a phylogeny suggested that baboon strains may have diverged prior to the clade containing human strains. We conclude that *T. pallidum* infection associated with genital lesions appears to be common in the wild baboons of the regions studied in Tanzania. Further study is needed to elucidate the infection's transmission mode, its associated morbidity and mortality, and the relationship between baboon and human strains.

## Introduction

In the 1960s and 1970s, researchers demonstrated that wild African primates were naturally infected with *Treponema pallidum*, the bacterium that causes syphilis (subsp. *pallidum*), yaws (subsp. *pertenue*), and bejel (subsp. *endemicum*) in humans. Serological surveys in West Africa showed that animals in many baboon troops were seropositive for *T. pallidum*, with seroprevalence surpassing 60% in certain areas of Senegal and Guinea [1], [2]. However, the clinical signs of infection described by researchers tended to be mild, when present at all, consisting of enlarged lymph nodes and small ulcers on the muzzle or near the armpit, which harbored high numbers of treponemes [3], [4].

The relationship between baboon and human strains in West Africa, where the majority of past studies have been conducted, has been poorly understood for decades. Investigators have noted that baboons living in regions in which humans had historically suffered from yaws or endemic syphilis appear to have a high seroprevalence of infection with *T. pallidum* themselves [1], [2], [5]. Humans can be artificially infected with baboon strains of *T. pallidum* [6], but in the wild it is unclear whether baboons serve as a reservoir for human infections, humans serve as a reservoir for baboon infection, or each host species harbors distinct strains [5]. Recently, a *T. pallidum* strain collected from a baboon in Guinea in the 1960s, known as the Fribourg-Blanc strain, was found to be more closely related to *T.p.* subsp. *pertenue* laboratory strains than to subsp. *pallidum* or *endemicum* strains [7]–[9]. It remains unclear whether this baboon strain will eventually be classified within the *pertenue* subspecies or whether it is closely related but distinct.

In contrast to the considerable number of studies in West African primates, research on *T. pallidum* infection in the wild primates of East Africa has been scant. Of 276 baboons captured in Kenya during the 1960s, none showed serological evidence of infection with *T. pallidum* [1]. This suggested that treponemal infection might be rare or non-existent in at least parts of East Africa, although yaws was present in this region well into the twentieth century [10]. However, in the late 1980s, researchers noticed a skin disease in several baboon troops at Gombe Stream National Park (GSNP), in Tanzania [11]. At this time, laboratory analysis of affected skin samples confirmed the presence of spirochetes by darkfield illumination, leading to the diagnosis of *T. pallidum* infection. Unlike the clinical signs noted in wild West African baboons previously, the disease at GSNP manifested in lesions in and around the genitals of both sexes. Because of the predilection for genital involvement and the observation that it appeared primarily in sexually mature animals, it was hypothesized that in this population of baboons, treponemal disease might be sexually transmitted [11]. Moreover, unlike the mild lesions described in West Africa, it was reported that lesions in a small portion of the individuals affected at GSNP became so severe that urinary flow was obstructed and death resulted [11]. In the 1990s, similar lesions were reported for the first time in the baboons at Lake Manyara National Park (LMNP) [12], [13], also in Tanzania but 700 km away.

Recently, Knauf *et al* [13] used a combination of macroscopic, histopathological, serological, and molecular biological methods to demonstrate that the infection causing genital-associated lesions in baboons at LMNP was caused by *T. pallidum*. Moreover, this study showed that many animals with no visible signs of disease tested positive for infection using molecular methods, including serology and PCR. Surprisingly, despite their ability to cause anogenital lesions similar to those present in human syphilis cases, the strains collected from LMNP baboons appeared most closely related to human *T. pallidum* subsp. *pertenue* (yaws-causing) strains.

Our motivation for this study was to clarify whether treponemal disease was present in baboons at various Kenyan and Tanzanian sites other than GSNP and LMNP, given the paucity of infection in this region suggested by a previous study [1], as well as to learn more about the strains active in East Africa. Specifically, our goals were to: 1) begin mapping the distribution of *T. pallidum* infection in wild baboons in the broader East Africa region; 2) further investigate the clinical manifestations of infection at LMNP; and 3) better characterize the strains circulating among baboons by sequencing six polymorphic regions in strains gathered from two different sites in Tanzania, LMNP and Serengeti National Park (SNP). We report that treponemal infection was found at the majority of Tanzanian sites examined but only one site in Kenya. Furthermore, we found that it is not uncommon for the disease to result in severe mutilation of the reproductive organs and that the strains responsible are genetically heterogeneous.

## Results

### Clinical manifestations consistent with *T. pallidum* infection at four sites in Tanzania

**Figure 1:**
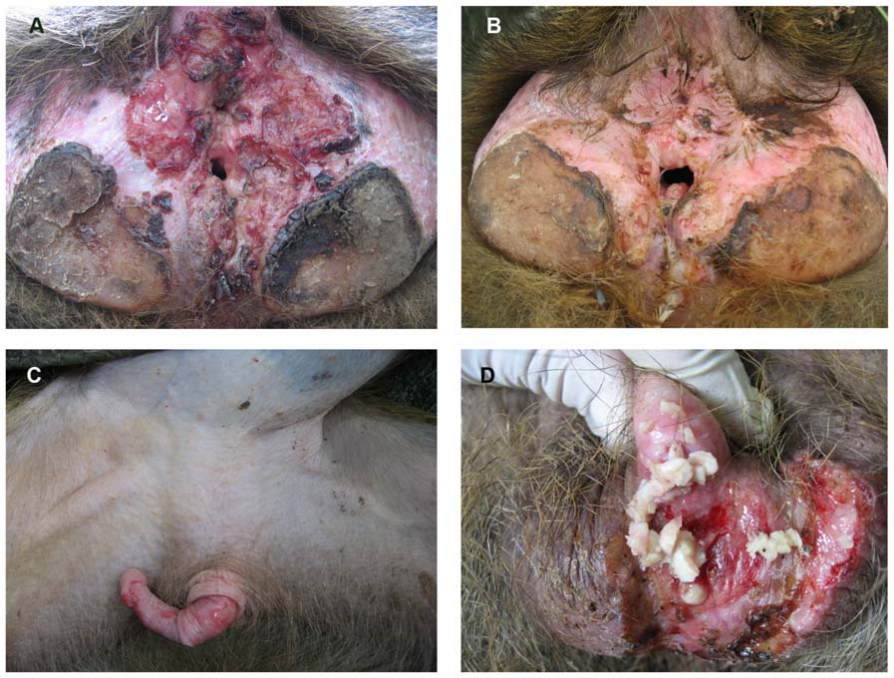
Gross pathology of olive baboons (*P. anubis*) with genital and circum-anal ulceration caused by *T. pallidum* at Lake Manyara National Park, Tanzania (2007). **A.** Adult female, severely affected, with massive destruction of the outer genitalia. The granulated tissue is fragile and bleeds easily on contact. The lesion is chronic and active. **B.** Adult female, severely affected, with massive destruction of the outer genitalia. The lesion is characterized by progressive scarification that has led to the vagina and anus being in a permanently open state. **C.** Sub-adult male, in an early stage of clinical infection. The corpus penis shows multiple erosions of the epidermis. **D.** Adult male with severe phimosis and genital ulceration. The prepuce is filled with smegma. The lesion is chronic and active.

Lesions in the anogenital region consistent with those described recently at Lake Manyara National Park [13], including a moderate to severe necrotizing dermatitis, were noted at all four Tanzanian sites visited in 2003–2004: Gombe Stream National Park (GSNP), Lake Manyara National Park (LMNP), Serengeti National Park (SNP), and Ngorongoro Conservation Area (NCA). The prevalence of genital ulceration ranged from 3.1% (or 8/256) of animals observed at SNP to 11.7% (or 32/273) at GSNP (**Table 1**). Photos representative of the moderate to severe lesions observed are presented in **Figure 1**.

**Table 1:** Sampling and site characteristics of baboon troops in which animals were tested for *T. pallidum*, including seroprevalence and the prevalence of outward signs of infection with *T. pallidum*.

Country	Site	Baboon Type	Years of Collection	Troops Observed	Outward Signs	Serum Sampling Strategy	Sample seroprevalence^a^
Tanzania	Gombe Stream National Park^b^	*Papio cynocephalus anubis*	2003–2004	4	32/273 (11.7%)	For the most part, sexually mature animals were sampled at random, although some animals with lesions were sampled purposively. A 1∶1 sex ratio was pursued.	5/8 (62.5%)
	Lake Manyara National Park	*Papio cynocephalus anubis*	2003, 2006	3	2003: 26/390 (6.7%). 2006: 41/300 (13.7%)	See above.	13/19 (68.4%)
	Serengeti National Park	*Papio cynocephalus anubis*	2003–2004	10	8/256 (3.1%)	See above.	12/25 (48.0%)
	Ngorongoro Conservation Area	*Papio cynocephalus anubis*	2003–2004	2	3/70 (4.2%)	See above.	N/A^c^
	Mikumi National Park	*Papio cynocephalus cynocephalus*	1983, 1985	1	0/44	All age and sex classes sampled.	0/44 (0.0%)
Kenya	Masai Mara National Reserve	*Papio cynocephalus anubis*	1985, 1987, 1993, 1994	7	0/63 (0.0%)	Adult males in troop sampled.	0/63 (0.0%)
	Amboseli National Park	*Papio cynocephalus cynocephalus*	1990, 2006	5	0/48 (0.0%)	Adult males and cycling females from five social groups sampled.	0/48 (0.0%)
	Mosiro	*Papio cynocephalus anubis*	1977	4	0/38 (0.0%)	Sampling excluded infants and nursing females.	0/38 (0.0%)
	Lake Magadi	*Papio cynocephalus anubis*	1977	3	0/18 (0.0%)	Sampling excluded infants and nursing females.	0/18 (0.0%)
	Gilgil	*Papio cynocephalus anubis*	1983	1	0/11 (0.0%)	Adult males in troop sampled.	0/11 (0.0%)
	Nanyuki	*Papio cynocephalus anubis*	1977	3	0/40 (0.0%)	Sampling excluded infants and nursing females.	23/40 (57.5%)

a Sample seroprevalence may be inflated at GSNP, LMNP, and SNP, as some baboons were purposively sampled in the serological survey. Three of eight animals in GSNP, seven of nineteen at LMNP, and five of twenty-five at SNP were included because they displayed anogenital lesions.

b GSNP has a history of antibiotic treatment of affected individuals [11], which may affect prevalence of infection there.

c The collection of biological samples was not permitted at this site.

During a second visit to LMNP in 2006, 300 sexually mature females and males, with a sex ratio of 1∶1, were examined in order to classify the frequency of severe disease manifestations. Up to 14.0% of animals of both sexes (14.0% females, 13.4% males) exhibited macroscopic lesions consistent with infection. In females, ulcerative lesions were frequently found around the genitals and anus, leading in some cases to a severe destruction and scarification of the outer genital structure (**Figure 1**). Seven females (4.7%) exhibited small ulcers on the genitals. Thirteen animals (8.7%) exhibited more widespread ulceration of the skin in the vaginal area, and one exhibited widespread ulceration of skin in both the vaginal and anal areas. In males, ulceration of penile tissue most often involved the tip of the glans penis, and it progressed towards the base of the penis (**Figure 1**). Four male baboons (2.7%) exhibited mild lesions on the genitals, four additional males (2.7%) experienced ulceration of the penile skin so severe that the tissue of the prepuce sloughed off, ten males (6.7%) lost the entire penis in this way, and two baboons lost the entire scrotum, including the testes. In total, fourteen females (9.3%) and sixteen males (10.7%) experienced major structural damage to the genitalia that appears to have resulted from infection with *T. pallidum*.

Throughout the two surveys, clinical signs of infection were never observed in juvenile baboons. Clinical signs were seen in subadult males who had begun to mate with female animals.

### Confirmation of anti-*T. pallidum* antibodies at three of the Tanzanian sites

Serum samples were taken at LMNP, GSNP, and SNP; unfortunately, no invasive sampling was permitted at NCA. Samples from all three of the affected sites were positive for *T. pallidum* antibodies using a very sensitive and specific immunochromatographic assay (**Table 1**). Three of eight samples were positive at GSNP (62.5%), 13 of 19 at LMNP (68.4%), and twelve of twenty-five at SNP (48.0%). Some of the animals sampled were chosen because they exhibited lesions consistent with the anogenital disease; purposively sampled baboons represented three of the five positive animals at GSNP, seven of the thirteen positive animals at LMNP, and five of the twelve positive animals at SNP.

### Serological assay for *T. pallidum* and observational reports at seven additional East African sites

Serum samples from one additional site in Tanzania (Mikumi National Park) and six sites in Kenya (Masai Mara National Park, Amboseli National Park, Mosiro, Lake Magadi, Gilgil, and Nanyuki) were examined using the very sensitive and specific *T. pallidum* particle agglutination test. Serological evidence of *T. pallidum* infection was found at only one of these sites, Nanyuki, where 57.5% of the animals tested positive for *T. pallidum* infection (**Table 1**). Field notes and observations by researchers at Mikumi and all of the Kenyan sites, including Nanyuki, did not include any mention of signs of an anogenital disease.

### Sequence Analysis and Phylogeny

**Figure 2:**
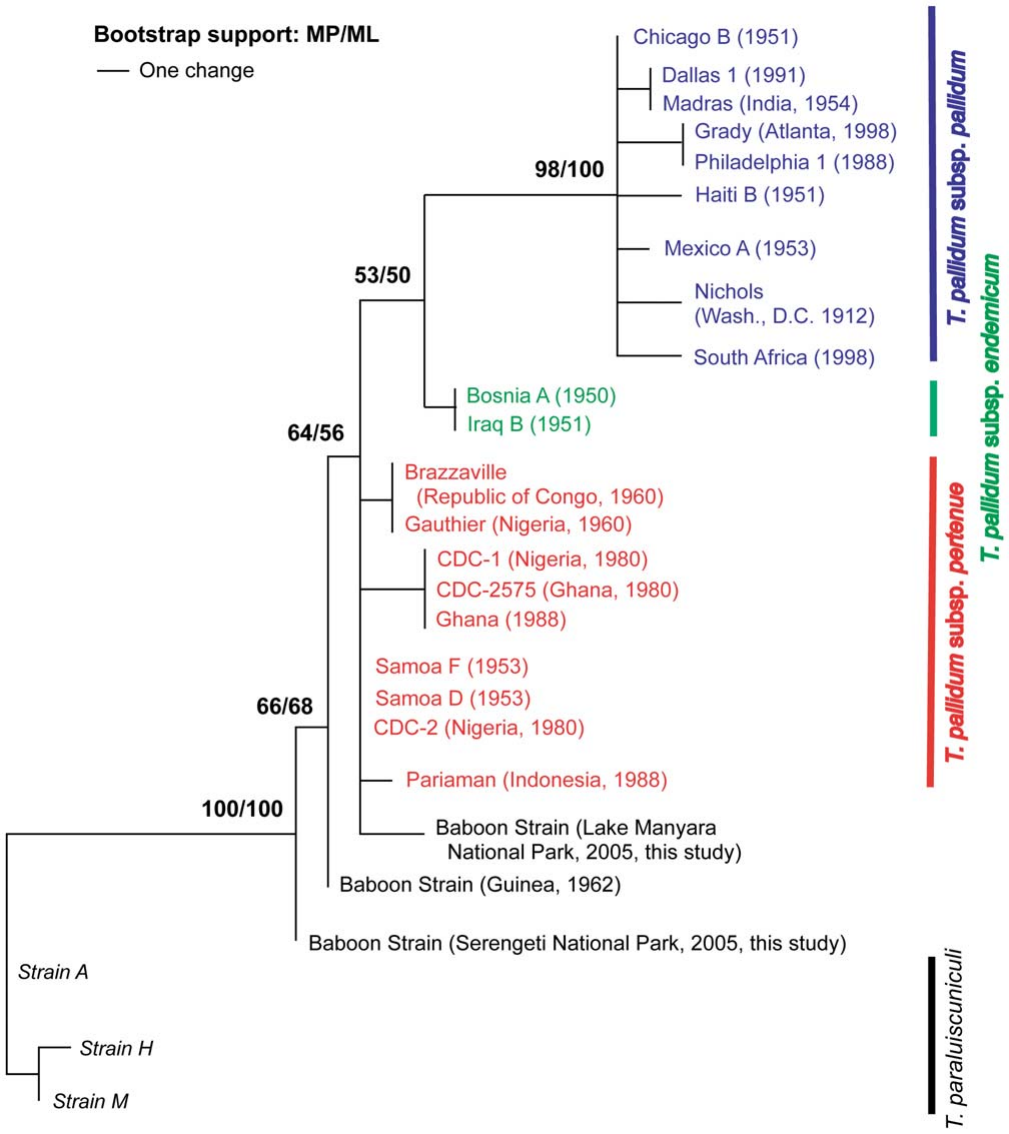
A phylogeny demonstrates that *T. pallidum* strains infecting baboons in Serengeti National Park and Lake Manyara National Park are genetically distinct from one another. Phylogenies were constructed using both Maximum Parsimony and Maximum Likelihood methods to analyze 25 polymorphisms in six concatenated regions of the *Treponema* genome. The phylogenies were congruent and a Maximum Parsimony tree was chosen for display, with bootstrap support displayed at all nodes that received greater than 50% using both methods.

In this study, DNA from one sample from SNP was successfully sequenced at six polymorphic regions: *deoD*, *gpd*, *tp92*, *tprI*, *cfpA*, and *tpF-1*. In addition, *tp92* and *cfpA* were sequenced in three samples from LMNP. These results, together with sequence data presented in Knauf *et al* [13], were used to build a phylogeny (**Table 2**, **Figure 2**) using Kimura's two-parameter model of nucleotide substitution. Both Maximum Likelihood and Maximum Parsimony methods were used, and the phylogenies they yielded were congruent. All three *T. pallidum* samples from LMNP were identical at the loci sequenced. The three distinct baboon strains included in the analysis clustered toward the base of the tree, although many of the bootstrap values on the tree were low (<70%). These strains included the ones collected from LMNP and SNP in Tanzania as well as the Fribourg-Blanc strain, which was collected in a well-studied area of Guinea in which no evidence of anogenital lesions had been reported [3]. While the SNP and Fribourg-Blanc strain appeared to diverge prior to the clade containing all human *T. pallidum* strains, the LMNP strain fell within a polytomy containing *T. p.* subsp. *pertenue* strains, which cause yaws, an infection of early childhood in humans. Although the genetic distance was shortest between the baboon strains and *T. p.* subsp. *pertenue*, the closest relative to the baboon strains among the human subspecies could not be determined in this analysis. It is noteworthy that despite being gathered at two sites relatively close to one another (∼170 km), the two Tanzanian baboon strains were genetically distinct, differing by four single nucleotide polymorphisms spread among four different genes. Trees constructed without the *tprI* and *tp92* genes, which contributed an inordinate number of polymorphisms (64%) to the alignment, were qualitatively similar to the one displayed here.

**Table 2:** Table of polymorphisms included in phylogenetic analysis.

*T. pallidum* subspecies	Strain^a^	*deoD*		*gpd*		*tp92*													*tprI*			*cfpA*			*tpF-1*	
**endemicum**	**Bosnia**	T	T	A	G	C	C	T	T	G	T	A	C	C	C	G	T	C	G	T	G	A	C	A	G	G
	**Iraq**	T	T	A	G	C	C	T	T	G	T	A	C	C	C	G	T	C	G	T	G	A	C	A	G	G
**pertenue**	**Brazzaville**	T	T	A	G	C	C	C	T	G	T	A	C	-	C	G	T	C	A	T	G	A	C	A	G	G
	**CDC1**	T	T	A	G	C	C	T	T	G	T	A	C	-	C	A	T	C	A	T	A	A	C	A	G	G
	**CDC2**	T	T	A	G	C	C	T	T	G	T	A	C	-	C	G	T	C	A	T	G	A	C	A	G	G
	**CDC2575**	T	T	A	G	C	C	T	T	G	T	A	C	-	C	A	T	C	A	T	A	A	C	A	G	G
	**Gauthier**	T	T	A	G	C	C	C	T	G	T	A	C	-	C	G	T	C	A	T	G	A	C	A	G	G
	**Ghana**	T	T	A	G	C	C	T	T	G	T	A	C	-	C	A	T	C	A	T	A	A	C	A	G	G
	**Pariaman**	T	T	A	G	C	A	T	T	G	T	A	C	-	C	G	T	C	A	T	G	A	C	A	G	G
	**Samoa D**	T	T	A	G	C	C	T	T	G	T	A	C	-	C	G	T	C	A	T	G	A	C	A	G	G
	**Samoa F**	T	T	A	G	C	C	T	T	G	T	A	C	-	C	G	T	C	A	T	G	A	C	A	G	G
**pallidum**	**Chicago B**	T	C	A	A	C	C	T	T	G	T	A	C	A	C	G	T	C	G	G	A	A	C	G	G	A
	**Dallas**	T	C	A	A	C	C	T	T	G	T	A	C	A	C	G	T	C	G	G	A	G	C	G	G	A
	**Grady**	T	C	A	A	C	C	T	G	G	T	A	C	C	C	G	T	C	G	G	A	A	C	G	G	A
	**Haiti B**	T	C	A	A	T	C	T	T	G	T	A	C	C	C	G	T	C	G	G	A	A	C	G	G	A
	**Madras**	T	C	A	A	C	C	T	T	G	T	A	C	A	C	G	T	C	G	G	A	G	C	G	G	A
	**Mexico A**	T	C	A	A	C	C	T	G	G	T	A	C	A	C	G	T	C	G	G	A	A	C	G	G	A
	**Nichols**	T	C	A	A	C	C	T	T	G	T	A	C	-	C	G	T	C	G	G	A	G	C	G	G	A
	**Philadelphia 1**	T	C	A	A	C	C	T	G	G	T	A	C	C	C	G	T	C	G	G	A	A	C	G	G	A
	**South Africa**	T	C	A	A	C	C	T	G	G	T	A	C	-	C	G	T	C	G	G	A	A	C	G	G	A
**N/A**	**TPC A**	C	T	G	G	C	C	T	T	A	T	A	C	-	-	G	C	T	-	-	-	A	T	A	A	G
**N/A**	**TPC H**	C	T	G	G	C	C	T	T	A	C	A	G	-	-	G	C	T	-	-	-	A	T	A	A	G
**N/A**	**TPC M**	C	T	G	G	C	C	T	T	A	T	A	G	-	-	G	C	T	-	-	-	A	T	A	A	G
**N/A**	**Baboon: SNP**	**C**	**T**	**A**	**G**	**C**	**C**	**T**	**T**	**G**	**T**	**A**	**C**	**-**	**C**	**G**	**T**	**C**	**A**	**T**	**G**	**A**	**C**	**A**	**A**	**G**
**N/A**	**Baboon: LMNP**	**T**	**T**	**A**	**G**	**C**	**C**	**T**	**T**	**G**	**T**	**G**	**C**	**-**	**C**	**G**	**T**	**C**	**A**	**T**	**A**	**A**	**C**	**A**	**G**	**G**
**N/A**	**Baboon: Guinea**	T	T	A	G	C	C	T	T	G	T	A	C	-	C	G	T	C	A	T	G	A	C	A	A	G
	**Synonymous or Nonsynonymous Substitution**	**S**	**S**	**S**	**S**	**N**	**N**	**N**	**N**	**S**	**N**	**N**	**N**	**N/D** b	**D**	**N**	**N**	**S**	**N**	**N**	**N**	**N**	**S**	**S**	**S**	**N**
	**Nucleotide Residue**	744	759	459	579	1592	1964	1966	1967	2010	2101	2209	2326	2388	2382–2399	2405	2408	2421	137	143	151	92	121	303	117	122

a TPC = *Treponema paraluiscuniculi* (agent that causes rabbit syphilis); SNP = Serengeti National Park; LMNP = Lake Manyara National Park.

b Deletion from nucleotide residues 2384–2398.

## Discussion

**Figure 3:**
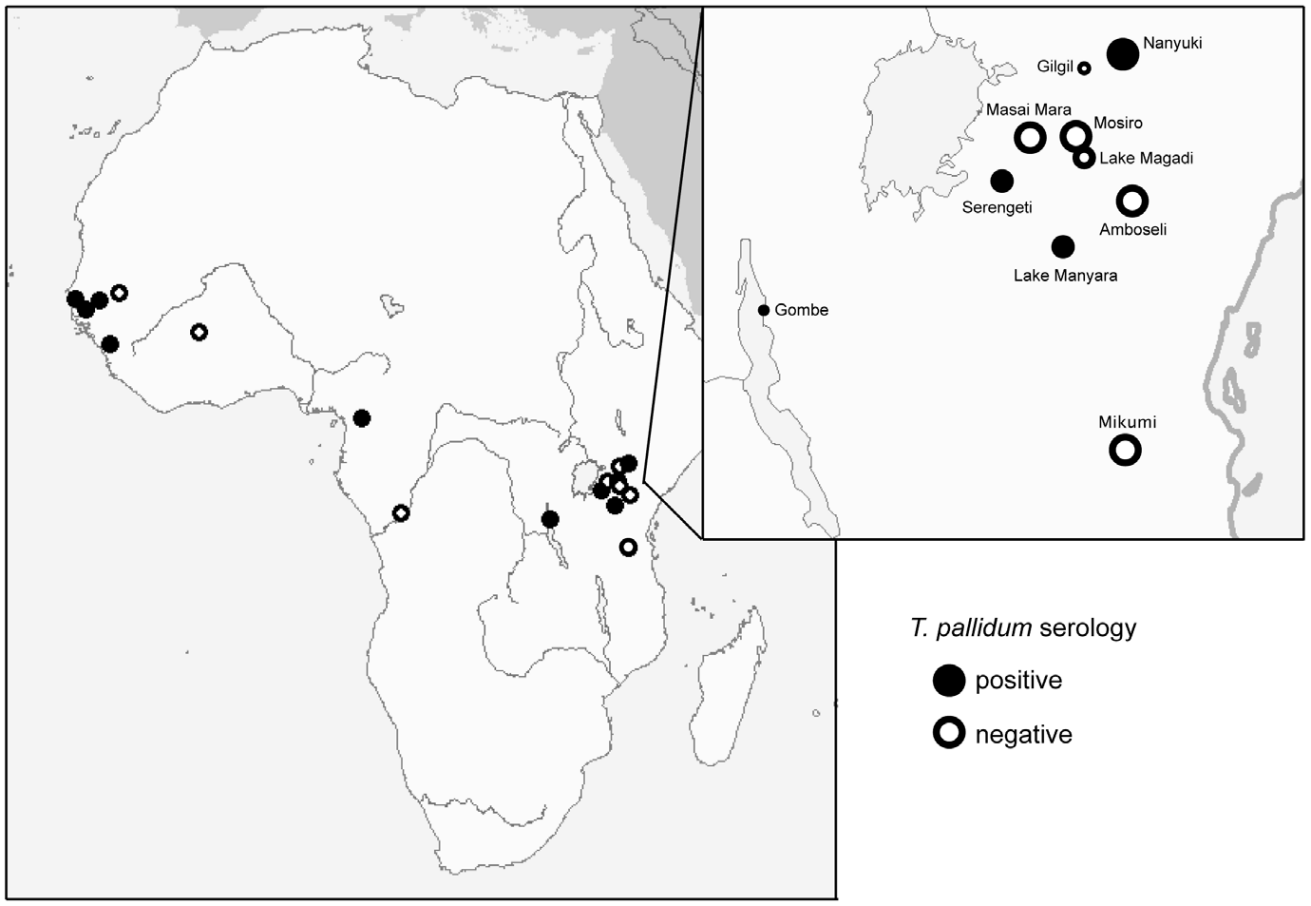
Geographic location of African sites where baboons have tested seropositive for *T. pallidum* antibodies. This map is based on the results presented in this paper (East African sites) as well as the results in [1],[2] (West African sites). Inset: East African sites examined in this paper, with circles proportional to the number of animals tested serologically at each site. Years of serum collection ranged from 1977–2006, as described in Table 1.

In this survey of wild East African baboons, we found that exposure to *T. pallidum*, confirmed via a highly sensitive and specific serological test, was common at all but one of the sites we investigated in Tanzania (**Figure 3**). By contrast, infection appeared to be absent at all but one of the sites examined in Kenya, supporting previous findings [1]. In addition, at all of the Tanzanian sites that exhibited positive serology, clinical signs of infection consistent with those described by Knauf *et al* [13], including necrotizing dermatitis that led to severe mutilation of the external genital organs, were noted (**Figure 1**). In 2006, we found that 9.3% of females (14/150) and 10.6% of males (16/150) at LMNP had suffered severe, macroscopically-evident disease-related damage to the genitalia, raising the possibility of significant infection-related morbidity and, perhaps in the most severe cases, even mortality.

Reports of genital-associated disease in nonhuman primates with a suspected link to *T. pallidum* first appeared in print in the late 1980s, in the form of a report on baboon health at GSNP [11]. Reports of a similar disease at LMNP surfaced in 1994 [12], and signs of the disease at other Tanzanian sites were not reported until the present study. Given these recent discoveries, we might expect this seemingly-unusual type of *T. pallidum* infection to be caused by a novel strain, perhaps one that is spreading in East African baboons. However, if a novel *T. pallidum* baboon strain emerged recently in Tanzania and spread rapidly across the country, one would expect isolates to be nearly identical genetically, especially given the low level of polymorphism typically found among strains of this bacterium [8], [14] and the small number of genes examined in this study. Instead, we found that the strains causing the disease in LMNP and SNP were genetically distinct, differing at four of 25 polymorphic sites used in the phylogeny (**Table 2**, **Figure 2**). While it is possible that recent, rapid evolution of the pathogen could explain the polymorphism, the fact that two synonymous substitutions in the LMNP strain are shared by human strains of ssp. *pertenue* as well makes this possibility seem unlikely, as it would require convergent evolution at sites that contain polymorphisms which are presumably neutral. It is more likely that the LMNP strain diverged from the SNP strain some time ago, and the former represents the non-human primate strain that shared the most recent common ancestor with human yaws-causing strains. Explanations that could potentially reconcile the high level of polymorphism between the *T. pallidum* strains responsible for the baboon disease in Tanzania with the seemingly recent appearance of the infection include a genetically heterogeneous bacterial population which seeded outbreaks at the sites studied, recent changes in host or environmental characteristics that fostered genital manifestations of the disease, or some combination of these factors.

The simian strain from LMNP could not be distinguished from human subsp. *pertenue* strains in our phylogeny (**Figure 2**). It falls within a polytomy that contains this baboon strain as well as all human subsp. *pertenue* strains. A relatively small number of polymorphic regions were examined in this study and bootstrap values at many of the nodes on our phylogeny were low (<70%); we expect that the position of this and other baboon strains relative to human strains may be clarified by sequencing additional polymorphic regions. If baboons are an important source of human infection, one might have expected human subsp. *pertenue* strains from West Africa to resemble the Fribourg-Blanc baboon strain collected in Guinea rather than other human subsp. *pertenue* strains from distant locations such as Samoa and Indonesia. Instead, all human *T. pallidum* strains appear to share a common origin. If further evidence demonstrates that the LMNP strain, like the other baboon strains, diverged prior to all human strains, and that the baboon strains form a paraphyletic group from which human strains are excluded, this will provide further support for the hypothesis that the *T. pallidum* strains that infect baboons have inhabited their hosts for a long period of time, are genetically diverse, and are distinct from human strains. Further, if *T. pallidum* strain phylogenies are congruent with host phylogenies, the evidence will indicate that this pathogen has evolved with its primate hosts, including humans, over millions of years.

While our study offers a preliminary overview of seroprevalence and manifestations of *T. pallidum* infection in the wild baboons of Tanzania and Kenya, there are some limitations. First, in order to investigate the prevalence of *T. pallidum* infection at all of the Kenyan sites and one of the Tanzanian sites (Mikumi), we relied on banked serum samples collected by various researchers over the years. Samples from a number of the sites, including Mikumi, Mosiro, Lake Magadi, Gilgil, and Nanyuki, were several decades old (**Table 1**). The temporal and spatial distribution of the disease may well be dynamic. Therefore, it is possible that sites that showed no evidence of infection at the time samples were gathered may be affected now, and it is also possible that baboons at Nanyuki, which showed a high prevalence of *T. pallidum* infection in 1977, may not be affected today. Second, the sampling scheme was not uniform at each site (**Table 1**), and, at many of the sites, reliable estimates for infection prevalence in the population as a whole could not be calculated. At GSNP, LMNP, and SNP, the number of animals from which serum was collected was relatively small, and at times animals with lesions were sampled purposively, which may have artificially inflated seroprevalence. In addition, a 1∶1 sex ratio was pursued when gathering serum samples at these sites, but in the typical baboon troop, which has a multi-male – multi-female social structure, females outnumber males. Finally, while positive serology provides a strong indication that a baboon has been exposed to *T. pallidum*, it does not necessarily mean that an animal is currently infected.

It is possible that the species of the baboon host has an effect upon the clinical manifestations and seroprevalence of infection. Previously, the only reports of *T. pallidum*-linked, genital-associated disease have been in olive baboons (*Papio anubis*) [11]–[13]. While other baboon species, such as the Guinea baboon (*Papio papio*), also carry the pathogen [1], their clinical signs, when present at all, have been described as small ulcers primarily on the muzzle [1], [2], [4], [15]. Perhaps genetic differences between species of baboons [16] influence susceptibility to the disease and/or lead to different clinical manifestations. In our study, each of the four Tanzanian sites affected by the genital disease was inhabited by olive baboons (**Table 1**). The single Tanzanian site that was not affected, Mikumi National Park, is exclusively inhabited by yellow baboons (*Papio cynocephalus cynocephalus*). It also lay further south than the other sites (**Figure 3**). Additional studies should help determine what role the host species plays in determining disease dynamics and presentation.

At present, there is no clear geographical trend differentiating affected and non-affected sites (**Figure 3**), and it is not apparent which factors govern the presence or absence of the infection in a given baboon population. Additional investigations that utilize both a standardized sampling procedure and a full spectrum of diagnostic methods for the detection of *T. pallidum* are needed, as in [13]. Careful examination of the characteristics of affected and non-affected baboon troops could shed light on risk factors for infection. The genital lesions associated with the disease and its predilection for sexually active animals suggest sexual transmission [11]. A careful study documenting the age of infection in affected troops as well as a thorough investigation of other factors that may lead to infection (e. g., vectors) could help determine if this is the dominant mode of transmission. Studies on the mode of spread, as well as spatial and temporal trends in disease prevalence, may help us better understand why infection is almost entirely limited to the Tanzanian side of the Kenyan/Tanzanian border; Masai Mara, a site where no evidence of the infection has been observed up to the present date, is geographically contiguous with SNP, a site that yielded abundant evidence of infection. Longitudinal studies could elucidate the level of morbidity and mortality associated with this infection. Given the damage to the reproductive organs associated with the disease (**Figure 1**) and evidence that even non-genital-associated *T. pallidum* lesions may affect reproductive success in gorillas [17], the possible deleterious effects of treponemal infection upon reproduction may be particularly important to examine in affected baboon populations. Finally, additional sequencing of *T. pallidum* strains collected from humans and baboons, as well as genetic characterization of *T. pallidum* strains in additional affected nonhuman primate species such as patas monkeys, gorillas, and chimpanzees [1], [18] could further clarify the relationship between human and nonhuman primate strains. If sexual transmission of the infection in baboons is demonstrated, then the strains responsible along with subsp. *pallidum* strains in humans may represent an example of the parallel evolution of sexual transmission in *T. pallidum*. By comparing the differences found between sexually and non-sexually transmitted baboon strains to the differences identified between subsp. *pallidum* strains and the non-sexually transmitted human subsp. *pertenue* and *endemicum* strains, it may be possible to learn more about the genetic basis of pathogenesis and sexual transmission in this pathogen.

In conclusion, this study demonstrates that a genital ulcer-associated *T. pallidum* infection in wild baboons is present at multiple sites in Tanzania and is linked to severe structural damage of the genitalia in a significant proportion of the animals observed. *T. pallidum* strains from two affected Tanzanian sites were compared and found to be genetically distinct. In addition, the phylogeny created in this study suggests that baboon strains may have diverged prior to human strains, but the low resolution of the tree prevents firm conclusions from being drawn. Clearly, further research is needed to understand the nature of *T. pallidum* infection in wild baboons. The importance of learning more about this disease becomes apparent when one considers 1) *T. pallidum*'s potentially deleterious effect upon other susceptible non-human primate species, such as chimpanzees [18]–[20] and gorillas [17], [21], [22]; and 2) the close proximity of some infected baboon troops to the habitat of these endangered species. Moreover, it will be essential to clarify the relationship between treponemal infections in humans and non-human primates, given the World Health Organization's plans to launch a second yaws eradication campaign [23].

## Materials and Methods

### Ethics Statement

The field investigations at Gombe Steam National Park (GSNP), Lake Manyara National Park (LMNP), Serengeti National Park (SNP), and Ngorongoro Conservation Area (NCA) were done by the Tanzania Wildlife Research Institute (TAWIRI), in accordance with TAWIRI's *Guidelines for Conducting Wildlife Research* (2001) and in compliance with Tanzania Veterinary Act number 16 of 2003 (Veterinary regulations; Government notice no. 389 of 2005), following a mandate of the Tanzania Wildlife Research Institute (TAWIRI) to conduct disease investigations in wildlife in different protected areas in Tanzania. The permission to conduct this research was granted by TAWIRI and the Tanzania Commission for Science and Technology (COSTECH). All biological samples were collected in order to perform research on treponemal disease in the animals residing there. In addition, *T. pallidum* DNA samples were gathered from baboons at LMNP in 2007, also as part of a research project on the genital associated disease, with the approval of the German Primate Center in Göttingen as well as TAWIRI, COSTECH, and Tanzania National Parks (TANAPA). Permission for both the observational studies and sampling in these protected areas was granted by the appropriate Tanzanian authorities (TAWIRI, COSTECH, and/or TANAPA).

Three-hundred and four additional serum samples from baboons examined in this study were collected at various sites in Kenya and Tanzania during the course of prior research studies. Fieldwork at Mikumi National Park was approved by the appropriate Animal Use Committees at Yale and Washington Universities as well as the Tanzanian National Scientific Research Council (now called COSTECH), TANAPA, and TAWIRI. Fieldwork at Masai Mara National Reserve and Gilgil was approved by the Animal Usage Committee of the Division of Laboratory Animal Medicine at Stanford University (Protocol # 9459) as well as the Office of the President and the Ministry of Tourism and Wildlife in Kenya. All protocols used at Amboseli National Park adhered to the laws and guidelines of Kenya (Kenya Research Permit numbers NCST 5/002/R/777 to SCA, and NCST 5/002/R/776 to JA) and were approved by the Animal Care and Use Committees at Duke University (A0840903) and Princeton University (1689) as well as the Office of the President and the Kenya Wildlife Service. Work at Mosiro, Lake Magadi, and Nanyuki was approved by the Northwestern institutional research compliance committees that oversaw animal care and use as well as the Office of the President and the Ministry of Tourism and Wildlife in Kenya. Permission to use all of the samples described in the current study was granted by the research groups who gathered them, all of which include authors of the current article. As specified above, each of these past studies had the permission of the appropriate authorities in the Kenyan or Tanzanian government as well as local landowners, where relevant.

Throughout each field study, animal welfare and ethical issues were taken into consideration during the immobilization of the baboons in order to minimize stress and discomfort, as described in greater detail for each site in the methods sections that follow.

Only data analysis was performed at Columbia University. Therefore, permission from an animal use committee was not required at this site.

### Field Investigations in Tanzania

A study of four protected areas in Tanzania was conducted from February 2003 to July 2004: GSNP, LMNP, SNP and NCA (**Table 1**). The study began with an observational investigation of the clinical signs of the disease and their prevalence. A baboon was considered to exhibit clinical signs of the disease if visible areas of genital ulceration were noted from a distance of 5 to 15 meters away. All baboons in the chosen areas were included in the counts, and some representative photographs were taken of the outward signs of infection. Additional photos were taken during follow-up surveillance visits to the sites.

Serum sampling was also conducted at GSNP, LMNP, and SNP at this time. For the most part, samples were collected at random from adults and subadults. However, a sex-ratio of 1∶1 was attempted when choosing animals for immobilization, and if a baboon with clinical signs of infection was encountered, it was purposively sampled. Baboons were captured using remote-distance injection via cold-gas tranquilizer-gun. Baboons were temporarily immobilized using a combination of ketamine hydrochloride (10 mg/kg) (Rotex Medica GmBH, Germany) and xylazine hydrochloride (0.5 mg/kg) (Kyron Laboratories (Pty) Ltd, South Africa) following a standardized protocol. Blood was collected from the femoral vein, and after separation serum was kept frozen in liquid nitrogen. In addition, a tissue biopsy was collected from an animal at SNP, using a 6-mm biopsy punch, and stored frozen in RNAlater Solution (Ambion, Inc., Germany) for subsequent DNA analysis. During the sampling procedure, animals were captured for only a short time and returned to their group afterwards without problems.

In November 2006, a second study was conducted at LMNP to investigate the prevalence and outward signs of advanced infection in more depth. A total of 300 adult and subadult baboons (the first 150 males and first 150 females encountered) were observed using binoculars. Animals were classified as 1) clinically unaffected (no visible signs of infection); 2) clinically affected, with mild to moderate outward signs of genital ulceration (lesions consistent with infection, including small ulcers on the genitals for males and females; or 3) clinically affected with advanced signs of infection (massive genital ulceration with severe, irreversible mutilation of genital structures).

In 2007, tissue biopsies were obtained from several baboons at LMNP, using the methods described above, and stored frozen in RNAlater for subsequent DNA analysis. Briefly, baboons were temporarily immobilized using a combination of ketamine hydrochloride (10 mg/kg) and xylazine hydrochloride (0.5 mg/kg) following a standardized protocol. The capture time was brief, and animals were immediately reunited with their group.

### Serum Samples from other East African Sites

In order to investigate the seroprevalance of *T. pallidum* among baboons in surrounding areas, 304 baboon sera collected from six sites in Kenya (Masai Mara National Park, Gilgil, Nanyuki, Mosiro, Lake Magadi, and Amboseli National Park) and one additional site in Tanzania (Mikumi National Park) were examined (**Table 1**). Samples had been gathered previously using different immobilization methods, which are described below, and kept frozen.

#### Mikumi National Park

Baboons were captured by darting or trapping. The animals were tranquilized with ketamine hydrochloride, given at 10 mg/kg of the animal's body weight. Blood was drawn from the femoral vein into vacutainer tubes containing either sodium heparin or EDTA. Blood was centrifuged in order to separate plasma within eight hours of collection, and plasma was frozen in liquid nitrogen.

#### Masai Mara National Reserve and Gilgil

Animals were treated humanely throughout the study, in accordance with National Institutes of Health guidelines. Individual baboons were darted using a blowpipe and anesthetized with phencyclidine HCL (approximately 2 mg/kg/body weight). Blood samples were collected within 15 minutes of darting.

#### Amboseli National Park

Individual baboons were captured one at a time using a hand-held blowpipe in an unobtrusive manner, with a dart bearing Telazol (4 mgl/kg), a combination of tiletamine and benzodiazepine. Blood was drawn from the saphenous vein into a serum separator tube, and serum was stored at ambient temperatures for 24–48 hours before being frozen. Darted animals were released when fully awake (after 3–4 hours) and rejoined their social group with no adverse effects. At this site, serum was diluted 1∶3 parts with RNAlater before being frozen. Control serum obtained from *T. pallidum*-reactive rabbits (Fujirebio Diagnostics, Malvern, PA), which was diluted in the same way and stored from 0–8 weeks, showed that this method of storage did not affect the test results obtained.

#### Mosiro, Lake Magadi, and Nanyuki

Baboons were captured using dropping-door traps baited with corn. The traps were visited at least twice daily, at which time any captured animals would be sedated and removed. Captured baboons were sedated with Sernylan (Parke Davis, discontinued) using a pole syringe, then brought to camp for blood collection. Baboons in this study were processed within 24 hours of capture and released after sampling.

### Serological Analysis

Sera obtained during the Tanzanian field studies were tested for *T. pallidum* antibodies in Tanzania, using the SYPHILITOP field kit (One Step Syphilis Test Strip, ALLDIAG, Strasbourg, France), which is a rapid chromatographic immunoassay with 97% sensitivity and 99% specificity. The other serum samples were analyzed in the United States, where the SYPHILITOP test is not available. These samples were analyzed using the Sero-DIA TPPA test (Fujirebio Diagnostics, Malvern, PA), which is a qualitative gelatin particle agglutination assay with 90% sensitivity and 100% specificity. Both tests were performed according to the manufacturer's instructions.

### DNA Analysis

DNA extraction was performed on the tissue biopsy samples from LMNP, as well as the tissue biopsy sample gathered from SNP in 2003–2004, using the Qiagen DNEasy Blood and Tissue Kit (Valencia, CA). Extraction was performed according to the manufacture's instructions.

Nested primers were constructed around 2,352 nucleotides in six polymorphic regions that have been shown to differentiate subsp. *pallidum*, *pertenue* and *endemicum* strains [8], [24]–[28] (**Table 3**). The primers were used to amplify these products in one sample from SNP and three from LMNP, the latter of which had been sequenced at four of the polymorphic regions in a previous study [13]. PCR amplifications were performed in 50 µL reactions containing 0.5 mM primers (Invitrogen, Carlsbad, CA), 200 µM GeneAmp dNTPs (Applied Biosystems, Foster City, CA), and 2.5 U AmpliTaq Gold polymerase with Gold Buffer and 3.0 mM MgCl_2_ (Applied Biosystems). PCR conditions were as follows: One cycle of 94° C for 5 minutes; 35 cycles of 94° C for 30 seconds, primer annealing at the appropriate temperature for 30 seconds, and 72° C for 1 minute and 30 seconds; followed by a final extension for seven minutes at 72° C. Standard precautions to avoid DNA contamination were employed. PCR products were Sanger sequenced by SeqWright (Houston, TX). The resulting sequences were deposited in GenBank under accession numbers EU683033, EU68035–EU683038, and FJ896488.

**Table 3:** Primers used in this study.

Gene	Primers (5′ to 3′)	Anneal. temp. °C	Product size (bp)	Source documenting polymorphisms
*tpf1* a	F:GAAAAAAATACCACAGCACCGC R:CAATGCCGTAGATGTGCCAGTG	57	171	[28]
*Gpd* a	F:AAGAACTTTCCCTCCTCCGTGC R:CGTTTGATACGCTTCAGCTCG	55	331	[26]
*deoD* a	F:GGTTACCAGAAAGGGCGTATTCC R:CGACCATTACACGACCATC	55	501	[27]
*cfpA* a	F:GAGTCCCAATGTGTTTCATCC R:GAACGCACACTTGACTACCG	55	556	[8], [24]
*tp92* a	F:AGAGCCTGAAGCTCGGGTAT R:ACCGTGAACGACAACACAAA	55	1030	[8], [25]
*tprI* b	F:CGTCACCCTCTCCTGGTAGT R:ATCCCTCGCCTGTAAACTGA	60	1830	[7], [8], [33]
*tpf1* Nested	F:CGTGCCATTGCTGCTATCT R:TGCCGTAGATGTGCCAGTG	56	100	
*gpd* Nested	F:GTGGGTTGGAACAGACAACC R:CGTTTGCACATACACTAGATCC	55	161	
*tp170* Nested	F:TAATGGCGTCCCCTTTGTTA R:GAAGCCACTACCGATGTGC	55	171	
*cfpA* Nested 1	F:GAGCGTCTGGACGTAATGG R:TAGGATGGCAATCTCCTTCG	55	189	
*cfpA* Nested *2*	F:CAATGTGTTTCATCCCGAAA R:CCTCCTTCGGCAGTTTAGTG	55	152	
*tp92* Nested	F:GGTGGGCTTTGACTTTGAAC R:TTTTCTTTGTTTTTGGGATGC	55	980	
*tprI* Nested	F:GTGAGAGGAGGGGGAGTGA R:CACCATTGGAAAGGAAGGAG	55	599	

a primers found in [8].

b primers found in [7].

### Phylogenetic Analysis

Polymorphisms which fell within regions in which no signature of recombination was detected were concatenated and aligned in ClustalX version 1.83 [29]. Recombination was ruled out using genomic searches for possible donor regions at highly polymorphic areas, utilization of the program RDP2, and analysis of dN/dS ratios to detect recombination events, as described previously [8]. All excluded polymorphisms were located in hypervariable regions of *tp92* and included nucleotide sites 1530–1606, 1801–1827, 2111–2135, and 2359–2373.

In Modeltest [30] the Akaike Information Criterion (AIC) was used to choose the appropriate model of nucleotide substitution. Phylogenetic trees were constructed in PAUP* [31] using Maximum Parsimony and Maximum Likelihood Methods, and *Treponema paraluiscuniculi*, the agent that causes rabbit syphilis, was used as an outgroup. Recently, some researchers have hypothesized that *T. paraluiscuniculi* evolved from a *T. pallidum*-like host [32], a possibility that would make it a poor phylogenetic outgroup. However, DNA sequences from the three ribosomal subunits of *T. paraluiscuniculi*, *T. pallidum*, and *Treponema denticola* (a bacterium that is more distantly related to the first two species) revealed identical sequences between *T. paraluiscuniculi* and *T. denticola* at all of the five informative polymorphisms identified (**Table S1**). Based on this evidence, we concluded that *T. paraluiscuniculi* is an appropriate phylogenetic outgroup. During tree-building, one thousand replicates were performed to obtain bootstrap support, and starting trees were obtained through random, step-wise addition. Tree bisection and reconnection was used for the branch-swapping algorithm. A Maximum Parsimony tree with branch lengths shown was chosen for display, with bootstrap support displayed at all nodes that received greater than 50% using both methods.

## Supporting Information

Table S1 *Treponema paraluiscuniculi* is identical to *Treponema denticola* ATCC 35405 at five informative genetic sites, while *T. pallidum* subsp. *pallidum* is identical to *T. denticola* at none.
(DOCX)
