# Correction: *Treponema pallidum* Infection in the Wild Baboons of East Africa: Distribution and Genetic Characterization of the Strains Responsible

**DOI:** 10.1371/journal.pone.0092489

**Published:** 2014-03-20

**Authors:** The PLOS ONE Staff

In Table 2, the sequence information for the Nichols and Mexico A strains at nucleotide position 2,388 of the tp92 gene was inadvertently switched. The correct sequence at this position is as follows: a deletion in Mexico A and an A in Nichols. Please see the corrected Table 2 here.

**Table 2:** Table of polymorphisms included in phylogenetic analysis.

*T. pallidum* subspecies	Strain^a^	*deoD*		*gpd*		*tp92*													*tprI*			*cfpA*			*tpF-1*	
**endemicum**	**Bosnia**	T	T	A	G	C	C	T	T	G	T	A	C	C	C	G	T	C	G	T	G	A	C	A	G	G
	**Iraq**	T	T	A	G	C	C	T	T	G	T	A	C	C	C	G	T	C	G	T	G	A	C	A	G	G
**pertenue**	**Brazzaville**	T	T	A	G	C	C	C	T	G	T	A	C	-	C	G	T	C	A	T	G	A	C	A	G	G
	**CDC1**	T	T	A	G	C	C	T	T	G	T	A	C	-	C	A	T	C	A	T	A	A	C	A	G	G
	**CDC2**	T	T	A	G	C	C	T	T	G	T	A	C	-	C	G	T	C	A	T	G	A	C	A	G	G
	**CDC2575**	T	T	A	G	C	C	T	T	G	T	A	C	-	C	A	T	C	A	T	A	A	C	A	G	G
	**Gauthier**	T	T	A	G	C	C	C	T	G	T	A	C	-	C	G	T	C	A	T	G	A	C	A	G	G
	**Ghana**	T	T	A	G	C	C	T	T	G	T	A	C	-	C	A	T	C	A	T	A	A	C	A	G	G
	**Pariaman**	T	T	A	G	C	A	T	T	G	T	A	C	-	C	G	T	C	A	T	G	A	C	A	G	G
	**Samoa D**	T	T	A	G	C	C	T	T	G	T	A	C	-	C	G	T	C	A	T	G	A	C	A	G	G
	**Samoa F**	T	T	A	G	C	C	T	T	G	T	A	C	-	C	G	T	C	A	T	G	A	C	A	G	G
**pallidum**	**Chicago B**	T	C	A	A	C	C	T	T	G	T	A	C	A	C	G	T	C	G	G	A	A	C	G	G	A
	**Dallas**	T	C	A	A	C	C	T	T	G	T	A	C	A	C	G	T	C	G	G	A	G	C	G	G	A
	**Grady**	T	C	A	A	C	C	T	G	G	T	A	C	C	C	G	T	C	G	G	A	A	C	G	G	A
	**Haiti B**	T	C	A	A	T	C	T	T	G	T	A	C	C	C	G	T	C	G	G	A	A	C	G	G	A
	**Madras**	T	C	A	A	C	C	T	T	G	T	A	C	A	C	G	T	C	G	G	A	G	C	G	G	A
	**Mexico A**	T	C	A	A	C	C	T	G	G	T	A	C	-	C	G	T	C	G	G	A	A	C	G	G	A
	**Nichols**	T	C	A	A	C	C	T	T	G	T	A	C	A	C	G	T	C	G	G	A	G	C	G	G	A
	**Philadelphia 1**	T	C	A	A	C	C	T	G	G	T	A	C	C	C	G	T	C	G	G	A	A	C	G	G	A
	**South Africa**	T	C	A	A	C	C	T	G	G	T	A	C	-	C	G	T	C	G	G	A	A	C	G	G	A
N/A	**TPC A**	C	T	G	G	C	C	T	T	A	T	A	C	-	-	G	C	T	-	-	-	A	T	A	A	G
N/A	**TPC H**	C	T	G	G	C	C	T	T	A	C	A	G	-	-	G	C	T	-	-	-	A	T	A	A	G
N/A	**TPC M**	C	T	G	G	C	C	T	T	A	T	A	G	-	-	G	C	T	-	-	-	A	T	A	A	G
N/A	**Baboon: SNP**	**C**	**T**	**A**	**G**	**C**	**C**	**T**	**T**	**G**	**T**	**A**	**C**	**-**	**C**	**G**	**T**	**C**	**A**	**T**	**G**	**A**	**C**	**A**	**A**	**G**
N/A	**Baboon: LMNP**	**T**	**T**	**A**	**G**	**C**	**C**	**T**	**T**	**G**	**T**	**G**	**C**	**-**	**C**	**G**	**T**	**C**	**A**	**T**	**A**	**A**	**C**	**A**	**G**	**G**
N/A	**Baboon: Guinea**	T	T	A	G	C	C	T	T	G	T	A	C	-	C	G	T	C	A	T	G	A	C	A	A	G
	**Synonymous or Nonsynonymous Substitution**	**S**	**S**	**S**	**S**	**N**	**N**	**N**	**N**	**S**	**N**	**N**	**N**	**N/D** b	**D**	**N**	**N**	**S**	**N**	**N**	**N**	**N**	**S**	**S**	**S**	**N**
	**Nucleotide Residue**	744	759	459	579	1592	1964	1966	1967	2010	2101	2209	2326	2388	2382-2399	2405	2408	2421	137	143	151	92	121	303	117	122

a TPC =  *Treponema paraluiscuniculi* (agent that causes rabbit syphilis); SNP = Serengeti National Park; LMNP = Lake Manyara National Park

b Deletion from nucleotide residues 2384-2

The incorrect information in Table 2 was used to create the phylogenetic tree in Figure 2. Please see the corrected Figure 2 here.

**Figure 2:**
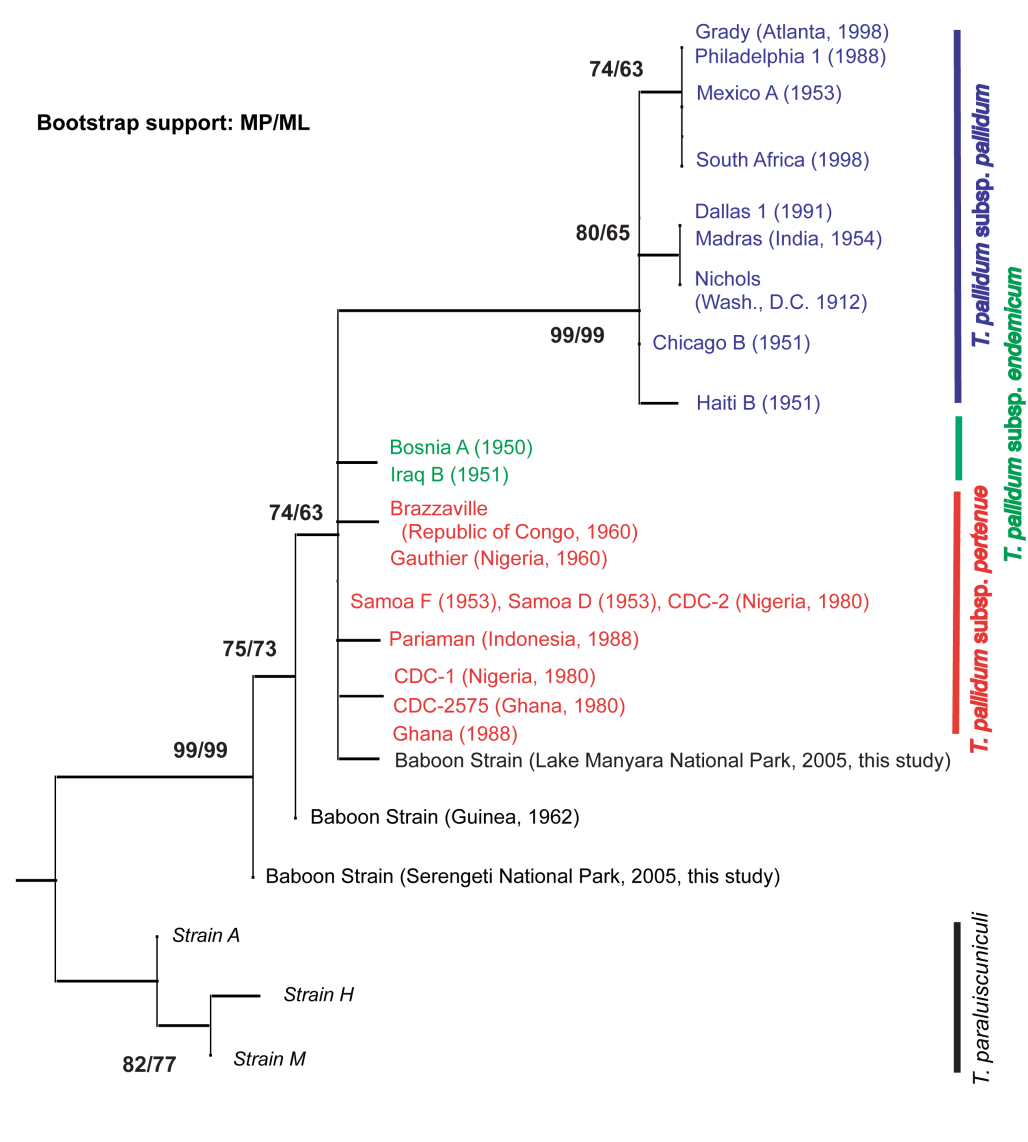
A phylogeny demonstrates that *T. pallidum* strains infecting baboons in Serengeti National Park and Lake Manyara National Park are genetically distinct from one another. Phylogenies were constructed using both Maximum Parsimony and Maximum Likelihood methods to analyze 25 polymorphisms in six concatenated regions of the *Treponema* genome. The phylogenies were congruent and a Maximum Parsimony tree was chosen for display, with bootstrap support displayed at all nodes that received greater than 50% using both methods.

The authors' explanation of the changes can be viewed on the Comments tab of the article page.
