# Supplementary material for: Treponema pallidum Infection in the Wild Baboons of East Africa: Distribution and Genetic Characterization of the Strains Responsible
Source: PLoS One. 2012 Dec 20;7(12):e50882. doi: 10.1371/journal.pone.0050882 (PMC3527465; doi:10.1371/journal.pone.0050882)
Supplement: Table S1 — Treponema paraluiscuniculi is identical to Treponema denticola ATCC 35405 at five informative genetic sites, while T. pallidum subsp. pallidum is identical to T. denticola at none. (DOCX) [file pone.0050882.s001.docx]

**Table S1**. *Treponema paraluiscuniculi* is identical to *Treponema denticola* ATCC 35405 at five informative genetic sites, while *T. pallidum* subsp. *pallidum* is identical to *T. denticola* at none.^a^

| **Ribosomal subunit** | ***Treponema* species** | **Number of sites shared with *T. denticola* ATCC 35405 and no other listed strain** | **Location of sites in gene** | **Strains and Accession Numbers** |
| --- | --- | --- | --- | --- |
| **5S** | *T. paraluiscuniculi* | **1** | A81C | Strain A (CP002103.1) |
|  | *T. pallidum* subspecies | 0 | N/A | subsp. *pallidum* [Dallas-1 (NC_016844.1), Chicago B (CP001752.1), Nichols, NC_000919.1), SS14 (NC_010741.1)], subsp. *pertenue* [CDC-2 (NC_016848.1), Samoa D (NC_016842.1), Gauthier (NC_016843.1)]  )]  ) |
| **16S** | *T. paraluiscuniculi* | **2** | A1134G  T1441C | Strain A (CP002103.1) |
|  | *T. pallidum* subspecies | 0 | N/A | subsp. *pallidum* [Dallas-1 (NC_016844.1), Chicago B (CP001752.1), Nichols, NC_000919.1), SS14 (NC_010741.1)], subsp. *pertenue* [CDC-2 (NC_016848.1), Samoa D (NC_016842.1), Gauthier (NC_016843.1)] |
| **23S** | *T. paraluiscuniuli* | **1** | G770A | Strain A (CP002103.1) |
|  | *T. paraluiscuniculi* and *T. pallidum* subsp. *pertenue* | **1** | A773G | Strain A (CP002103.1), subsp. *pertenue* [CDC-2 (NC_016848.1), Samoa D (NC_016842.1), Gauthier (NC_016843.1)] |
|  | *T. pallidum* subsp. *pallidum* | 0 | N/A | subsp. *pallidum* [Dallas-1 (NC_016844.1), Chicago B (CP001752.1), Nichols, NC_000919.1), SS14 (NC_010741.1)] |

^a^ Identical results for ribosomal RNA operons 1 and 2
